# Toward a Model of Age Inclusivity in Higher Education

**DOI:** 10.1093/geroni/igab046.040

**Published:** 2021-12-17

**Authors:** Susan Whitbourne

**Affiliations:** University of Massachusetts Boston, Boston, Massachusetts, United States

## Abstract

The AFU principles clearly state the aspiration of promoting age inclusivity in higher education within the context of the UN Sustainability Development Goals. With these principles as a starting point, the Age-Friendly Campus Climate Inventory and Survey were developed to assess the extent to which AFU principles are put into practice (Inventory) and how campus constituencies perceive these practices. Based on social ecological models, a framework for measuring age inclusivity was developed in which practices ("objective environment") are compared to perceptions ("subjective environment"). Participating campuses (N=29) completed the inventory for each major executive unit, providing scores that were grouped by major campus functions, including research, teaching, community engagement, and support. By comparing these scores with perceptions of each function by samples of constituencies of faculty, staff, and students, it is possible to test the person-environment match as conceptualized by social ecological models providing important clarification for the AFU principles.

